# Diagnostic reference levels for indication-based CT categories in pediatric CT: data from an international registry

**DOI:** 10.1007/s00330-025-11724-9

**Published:** 2025-06-14

**Authors:** Denise Bos, Yifei Wang, Carly Stewart, Philip W. Chu, Jason Luong, Johannes Haubold, Benjamin Schröer, Sebastian Zensen, Phuong-Anh T. Duong, Sebastian Schindera, Cécile R. L. P. N. Jeukens, Marco Das, Andrew J. Einstein, Luisa Cervantes, Rebecca Smith-Bindman

**Affiliations:** 1 Institute of Diagnostic and Interventional Radiology and Neuroradiology, University Hospital Essen, University Duisburg-Essen, Essen, Germany; 2 Institute of Diagnostic and Interventional Radiology, University Hospital Zurich, Zurich, Switzerland; 3 Department of Epidemiology and Biostatistics, University of California San Francisco, San Francisco, CA USA; 4 Faculty of Medicine, University Duisburg-Essen, Essen, Germany; 5 Department of Radiology, NYU Grossman School of Medicine, New York, NY USA; 6 Institute of Radiology, Cantonal Hospital Aarau, Aarau, Switzerland; 7 Department of Radiology and Nuclear Medicine, Maastricht University Medical Centre+, Maastricht, The Netherlands; 8 Department of Diagnostic and Interventional Radiology, Helios Rhein-Ruhr Kliniken GmbH, Duisburg, Germany; 9 Seymour, Paul, and Gloria Milstein Division of Cardiology, Department of Medicine, and Department of Radiology, Columbia University Irving Medical Center/NewYork-Presbyterian Hospital, New York, NY USA; 10 Department of Radiology, Nicklaus Children’s Hospital, Miami, FL USA; 11 Department of Obstetrics, Gynecology and Reproductive Sciences, University of California San Francisco, San Francisco, CA USA; 12 Philip R. Lee Institute for Health Policy Studies, University of California San Francisco, San Francisco, CA USA

**Keywords:** Tomography (X-ray computed), Pediatrics, Diagnostic imaging, Diagnostic reference levels, Registries

## Abstract

**Objectives:**

Computed tomography (CT) radiation doses vary depending on medical indications, protocols, and local practice. Our aim is to establish diagnostic reference levels (DRLs) for CT categories based on clinical indications in pediatric patients.

**Materials and methods:**

We analyzed CT data from an international dose registry retrospectively, including scans performed in children under 18 years at 143 facilities between January 2016 and January 2021. DRLs were calculated for volumetric CT dose index (CTDI_vol_), dose-length product (DLP), and size-specific dose estimate (SSDE) across 14 CT categories, which reflect both the anatomic areas and radiation dose levels (low, routine, high dose) required by imaging indications within the anatomic areas. We compared the routine dose categories for head, chest, and abdomen/pelvis scans between facilities in the United States (U.S.) and Europe.

**Results:**

A total of 95,047 CT scans (mean age, 10.4 ± 5.6 years, 54% male), including 41 different indications, were included. DRLs increased with increasing age group (*p* < 0.05 for trend). For the head and the abdomen/pelvis body regions, there was greater variation between the CT categories than between the indications within the categories, suggesting these categories are valid. For example, in 10- to 15-year-old children, the DRLs for the DLP increased from 362 mGy·cm for head low dose to 2058 mGy·cm for head high dose. The U.S. DRLs were similar to the European for head categories, but were around twice as high for routine dose chest, and abdomen/pelvis.

**Conclusions:**

We have established DRLs for indication-based CT category age groups to help standardize practice.

**Key Points:**

***Question*** *CT categories are needed to reflect body regions and clinical indications with similar radiation dose requirements in pediatric patients*.

***Findings*** *Doses varied more between CT categories than within. DRLs for chest and abdomen/pelvis were about twice as high in the U.S. as in Europe*.

***Clinical relevance*** *DRLs as a function of patient age were developed for 14 broad anatomy- and indication-based CT categories in children using registry data*.

**Graphical Abstract:**

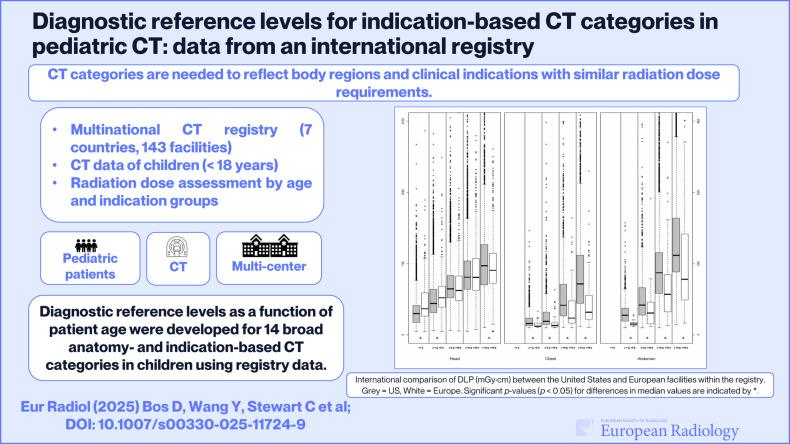

## Introduction

After an increase in pediatric CT in the United States (U.S.) in the early 2000s, growth in utilization had stabilized or declined by 2016, yet overall use remains high, with around 3–9 million examinations performed annually in children in 2022–2023 [1–6].

Radiation exposure from CT is an important concern in pediatric patients because of its greater carcinogenic risk in children compared with adults [7–11]. For every 10,000 children examined with CT, at least one to two children will develop a hematologic malignancy and one child will develop a brain cancer in a follow up period of 12 years (hematologic malignancy) and 5–15 years (brain cancer), estimated and reported by the European EPI-CT study [12, 13]. Thus, CT radiation doses should be optimized to minimize the potential harms relative to the value of imaging.

Diagnostic reference levels (DRLs) are identified as commonly used levels of radiation dose for specific examination types that can serve as benchmarks for radiation dose optimization, allowing comparison of commonly applied radiation exposures between different protocols, CT scanners, and institutions [14]. However, only a small number of countries have established DRLs for pediatric examinations, and for many types of examinations, there are no national DRLs available [15].

Radiation dose varies widely across different CT protocols and clinical indications for imaging [16]. While some indication-based DRLs have been established for adults [17, 18], in children, DRLs are typically established only for routine CT examinations of the head, chest, and abdomen/pelvis and are not indication-based [15, 19]. The widest range of pediatric DRLs has been reported for the ten most commonly performed CT examinations in children in the U.S., but they reflect the CT protocol rather than the indications for the examination [6]. Most useful are DRLs by groups of clinical indications that have the same dose and image quality requirements, which provide easier comparisons due to a smaller set of doses compared to protocol-based ones. Such indication-level DRLs have been established for adults [17, 20], but are missing in children.

The purpose of this study is to establish DRLs for children based on CT indications and anatomical area and to group the indications into CT categories, where each category includes indications with similar dose and image quality requirements, similar to the approach previously taken in adults using data from the University of California San Francisco (UCSF) CT International Dose Registry [20].

## Materials and methods

### UCSF CT International Dose Registry (Registry)

The study was approved by the UCSF Committee on Human Research, and contributing sites either relied on UCSF approval or obtained local institutional review board approval. Written informed consent was waived. Between January 1, 2016 to January 1, 2021, the Registry collected diagnostic CT data on children under 18 years of age from 143 facilities at 23 sites in seven countries (U.S., Switzerland, Netherlands, Germany, United Kingdom, Israel, Japan) using Radimetrics^TM^ dose management software (Bayer) and CT data were retrospectively analyzed. Detailed information and results have been published previously [16, 17]. Data were derived from 321 CT scanners, including 71 models from the four largest manufacturers: GE HealthCare Technologies Inc., Philips, Siemens Healthineers, and Canon/Toshiba Corporation, Table 1.

**Table 1:** Characteristics of included patients and CT examinations

Characteristic	*N*	Percent*
Total	95,047	100.0%
Age group		
Under 1-year-olds	6495	6.8%
1- to under 5-years-olds	12,905	13.6%
5- to under 10-year-olds	16,961	17.8%
10- to under 15-year-olds	27,124	28.5%
15- to under 18-year-olds	31,562	33.2%
Sex		
Male	51,651	54.3%
Female	42,935	45.2%
Other sex	342	0.4%
Missing sex	119	0.1%
Geographic region		
USA	87,723	92.3%
Europe	4195	4.4%
Israel	2465	2.6%
Japan	664	0.7%
Scanner manufacturer		
Siemens	46,625	49.1%
GE	38,931	41.0%
Canon	5755	6.1%
Philips	3497	3.7%
Hitachi	237	0.2%
Missing manufacturer	2	0.0%
Body region		
Head	49,622	52.2%
Spine	10,005	10.5%
Cardiac	316	0.3%
Chest	7179	7.6%
Abdomen and pelvis	20,843	21.9%
Extremity	4875	5.1%
Combined	2207	2.3%

* Percentages may not sum to 100% due to rounding

### Indication-based CT categories

CT examinations were categorized into groups based on body region and clinical indication as determined by using natural language processing (NLP), a computational method for analyzing and interpreting human language. NLP was applied to the study description and protocol names found in the digital imaging and communications in medicine (DICOM) header data, an approach found to be 90% accurate compared with expert chart review [20]. Most examinations could be assigned to a body region without detailed information on the clinical reason or indication for the scan, and were grouped as NOS (not otherwise specified). A total of 14 CT categories were created, reflecting anatomic region and radiation dose level (Table 2).

**Table 2:** CT indications associated with CT categories for head, spine, cardiac, chest, abdomen and pelvis, extremity, and combined imaging

Body region	Radiation dose level		
	Low	Routine	High
Head	Sinus	Head not otherwise specified	Head and neck perfusion angiography
	Temporal bone	Trauma	Head perfusion angiography
	Facial bones, cranial floor	Suspected hemorrhage, suspected stroke	
Spine		Neck	
		Cervical spine	
		Neck angiography	
		Thoracic spine	
		Combined thoracic/lumbar spine	
		Lumbar spine	
Cardiac		Cardiac not otherwise specified	
		Coronary angiography	
Chest		Chest not otherwise specified	
		Pulmonary embolism	
		Angiography	
		Noncontrast chest	
		High-resolution chest	
		Dissection	
Abdomen and pelvis		Abdomen not otherwise specified	Urogram
		Angiography	Acute gastrointestinal bleeding
		Enterography	Angiography for aortic injury
		Suspected appendicitis	Liver
		Suspected kidney stones	
		Pain, weight loss, suspected mass, nausea, vomiting, diarrhea	
		Abscess	
		Bladder without contrast	
		Bladder with contrast	
Extremities		Upper extremity	Upper extremity angiography
		Lower extremity	Lower extremity angiography
Combined		Combined chest and abdomen	
		Whole body	

### CT phantom type

Radiation dose metrics can only be compared if they are used with the same phantom for calculating the volumetric CT dose index (CTDI_vol_). Head CT examinations using the 16 cm phantom, head and neck perfusion examinations using both the 16 cm and 32 cm phantoms, and body examinations using the 32 cm phantom were included. The phantom type used was extracted from the dose monitoring software. Examinations with an unspecified or missing phantom type or that used the “wrong” (less commonly used) phantom type were excluded [21, 22], resulting in the exclusion of 6300 examinations (6.2% of 101,347).

### Age groups

Patients were grouped into five age groups (under 1 year, 1 year to under 5 years, 5 years to under 10 years, 10 years to under 15 years, 15 years to under 18 years). The age group definition does not represent the somatic development of children, but was used as commonly used in the international literature to define DRLs.

### Dose metrics and scan length

The dose metrics reported are the CTDI_vol_, the dose-length product (DLP), and the size-specific dose estimate (SSDE) from all imaging acquisitions of the CT examination, including bolus scans. The SSDE provides a more precise assessment of the radiation dose received by a patient than CTDI_vol_, as it is adjusted based on the patient’s size [23]. Patient diameter, as a measure of patient size, was provided by Radimetrics^TM^ dose management software, and is defined as the effective diameter of the anatomic area being examined. The effective diameter is calculated as the square root of the product of the anterio–posterior and lateral diameters. It is also used to determine the appropriate radiation dose required for an examination and to calculate SSDE. In this study, the effective diameter of each series is calculated for the mid-slice of each scan region. Thereafter, the diameter of an exam was defined as the mean effective diameter of its constituent series. The examination-level CTDI_vol_ and SSDE are respectively defined as the average CTDI_vol_ and SSDE of the examination’s constituent series, weighted by scan length. This weighting was performed to consider the influence of scan length on the overall average radiation dose metrics. The examination-level DLP is defined as the sum of the examination’s constituent series. The SSDE is not reported for extremity and head categories due to nearly complete missing data. For all other anatomical regions, SSDE values were not available for between 4.7% (cardiac) and 16.8% (spine) of examinations. Radiation doses for age groups with a sample size of less than ten examinations per CT category were not reported. The reported scan length is the mean scan length of all diagnostic CT series in one examination.

### International comparison

The radiation doses for the routine dose head, chest, and abdomen and pelvis categories were compared between the U.S. and Europe. Insufficient sample size precluded comparison with Japan and Israel.

### Statistical analysis

For each indication and CT category, the DRL was defined as the 75th percentile of the dose distribution, as proposed by the International Commission on Radiological Protection Report 135 [24]. The achievable dose (AD) was defined as the median of the dose distribution. The 95% confidence intervals (CI) of the DRLs and ADs were calculated by bootstrapping using random sampling with replacement. Bootstrapping was used to determine if the DRLs and ADs varied by anatomic region, age group, and geographic region. A *p*-value < 0.05 was considered statistically significant. Analyses of variance were performed for the DLP to assess if there was greater variation between CT categories of the same anatomic area than between indications within the same category to validate the CT categories and the assignment of CT indications to categories. For example, in the head, if there was greater variation between the low, routine and high dose head categories, than between the individual indications within the categories (e.g., within the low dose categories, the indications of facial bones/cranial floor, temporal bones, and sinus indications), than the three dose categories would be supported [20]. This analysis was limited to the subdivided body regions (head, and abdomen and pelvis where there were multiple dose levels and multiple indications within dose level). Analyses were done using R version 4.2.1.

## Results

A total of 95,047 CT examinations were included. The mean age was 10.4 ± 5.6 years, 54.3% were performed in males, and 33.2% were performed in the oldest age group, Table 1. The included data were primarily from the U.S. (92.3%), followed by European institutions (4.4%). The majority of CT scans (52.2%) were performed on the head, followed by the abdomen and pelvis (21.9%). CT scans were primarily performed on Siemens CT scanners (49.1%), followed by GE (41.0%), Table 1. Examinations were grouped into 14 broad CT categories reflecting 41 CT indications, Table 2. The sample size for each of the CT categories varied greatly, with some categories infrequently represented (e.g., L-spine in young children, head high dose) and others having many thousands of children (e.g., routine dose head and abdomen and pelvis), Table 3. The number of facilities contributing data for a CT category ranged from 6 to 127 (mean 73 ± 45 facilities), and the number of CT scanner models ranged from 4 to 47 (mean 31 ± 15).

**Table 3:** Radiation doses by CT category and age group

CT category	Age group (years)	No. of CT examinations	DLP (mGy·cm)		Volumetric CT dose index (CTDI_vol_ in mGy)		SSDE (mGy)	
			Median (95% CI)	DRL (95% CI)	Median (95% CI)	DRL (95% CI)	Median (95% CI)	DRL (95% CI)
Head low dose	< 1	330	107 (96–119)	223 (195–249)	9.1 (8.2–9.8)	15.7 (14.0–17.7)	–	–
	1 to < 5	1192	178 (168–188)	277 (267–296)	10.0 (9.8–10.6)	21.1 (20.8–21.6)	–	–
	5 to < 10	2736	199 (195–206)	298 (288–311)	12.5 (12.3–13.0)	25.8 (24.1–26.8)	–	–
	10 to < 15	3510	250 (245–256)	362 (347–376)	14.7 (14.4–15.0)	26.7 (25.8–27.3)	–	–
	15 to < 18	3193	285 (281–291)	461 (442–484)	16.3 (15.8–16.7)	28.0 (27.2–28.2)	–	–
Head routine dose	< 1	5406	211 (209–214)	292 (287–297)	14.0 (13.8–14.3)	18.2 (17.9–18.4)	–	–
	1 to < 5	8040	316 (309–321)	453 (445–459)	18.0 (17.4–18.5)	24.1 (23.7–24.5)	–	–
	5 to < 10	7227	453 (449–456)	585 (579–592)	26.0 (25.8–26.1)	31.3 (30.9–31.6)	–	–
	10 to < 15	8980	571 (562–578)	734 (726–742)	30.9 (30.6–31.1)	38.3 (37.9–38.6)	–	–
	15 to < 18	8965	683 (677–690)	895 (882–910)	36.4 (36.1–36.7)	46.0 (45.4–46.7)	–	–
Head high dose	< 1	0	–	–	–	–	–	–
	1 to < 5	3	–	–	–	–	–	–
	5 to < 10	6	–	–	–	–	–	–
	10 to < 15	19	1304 (1028–1813)	2058 (1324–2536)	59.5 (45.2–115.3)	123.2 (63.2–286.2)	–	–
	15 to < 18	15	2306 (1673–2941)	3115 (2275–4354)	109.9 (73.0–264.5)	268.4 (109.9–394.0)	–	–
C-Spine	< 1	130	56 (53–64)	99 (78–139)	3.3 (3.1–3.7)	4.5 (4.2–4.8)	8.7 (7.4–9.7)	11.0 (10.0–11.4)
	1 to < 5	1073	71 (69–73)	94 (90–96)	3.8 (3.6–3.9)	4.6 (4.5–4.7)	9.8 (9.4–10.0)	11.4 (11.3–11.7)
	5 to < 10	1303	98 (94–101)	137 (128–147)	4.6 (4.5–4.7)	5.7 (5.5–5.9)	11.0 (10.7–11.3)	13.5 (13.2–13.8)
	10 to <15	1870	180 (173–186)	273 (264–284)	7.4 (7.0–7.6)	11.3 (11.0–11.6)	16.2 (15.4–16.9)	25.2 (23.6–26.5)
	15 to < 18	2268	260 (251–266)	390 (377–403)	10.0 (9.7–10.3)	15 (14.4–15.4)	21.1 (20.5–21.8)	35.5 (34.1–36.9)
T-Spine	< 1	5	–	–	–	–	–	–
	1 to < 5	26	84 (68–121)	133 (92–145)	2.6 (2.1–3.2)	3.2 (2.7–3.8)	6.5 (4.8–8.0)	8.1 (6.7–9.6)
	5 to < 10	119	115 (98–136)	185 (157–219)	3.4 (3.1–3.8)	5.3 (4.3–6.1)	7.6 (6.4–8.5)	12.0 (10.0–13.5)
	10 to < 15	484	223 (215–241)	346 (327–364)	5.8 (5.2–6.1)	9.0 (8.3–10.2)	10.5 (9.2–11.3)	18.2 (16.4–19.6)
	15 to < 18	411	375 (346–398)	589 (557–681)	8.6 (7.8–9.6)	14.1 (12.7–15.2)	15.9 (14.8–18.2)	25.9 (24.1–27.6)
L-Spine	< 1	12	492 (75-–1204)	1221 (454–1496)	16.0 (2.8–29.9)	26.6 (15.3–53.5)	20.3 (2.8–64.3)	43.6 (8.7–182.5)
	1 to < 5	32	71 (55–97)	119 (79–131)	2.2 (1.3–3.1)	3.4 (2.7–4.8)	3.9 (2.9–7.9)	8.2 (5.9–11.9)
	5 to < 10	106	98 (86–117)	167 (130–235)	3.5 (3.0–4.1)	5.9 (4.9–6.7)	8.1 (6.8–9.1)	13.2 (10.8–14.7)
	10 to < 15	712	182 (170–194)	302 (284–325)	10.1 (9.7–10.8)	15.0 (14.7–15.3)	21.3 (20.0–22.7)	33.0 (31.1–33.7)
	15 to < 18	1454	319 (307–332)	491 (471–527)	14.0 (13.5–14.5)	18.6 (18.1–19.5)	27.5 (26.3–28.5)	38.4 (38.3–39.9)
Cardiac	< 1	38	42 (33–47)	49 (46–54)	1.3 (0.9–1.4)	1.4 (1.4–1.8)	3.1 (2.2–3.3)	3.3 (3.2–3.8)
	1 to < 5	56	49 (44–57)	64 (57–74)	1.5 (1.4–1.6)	1.7 (1.6–1.9)	3.2 (3.1–3.3)	3.6 (3.3–3.8)
	5 to < 10	55	49 (43–57)	75 (57–93)	1.9 (1.9–3.0)	3.6 (3.0–4.5)	4.4 (3.8–5.8)	6.9 (5.8–7.5)
	10 to < 15	91	80 (63–89)	116 (96–144)	3.9 (3.4–4.0)	5.4 (4.5–6.9)	6.5 (5.9–7.5)	9.8 (8.0–12.7)
	15 to < 18	76	128 (98–156)	176 (160–230)	5.5 (4.1–6.5)	8.1 (6.8–10.0)	8.7 (7.3–10.9)	13.8 (11.0–19.1)
Chest	< 1	230	44 (41–47)	76 (63–84)	2.3 (2.0–2.4)	3.6 (3.2–4.5)	4.8 (4.4–5.4)	8.1 (6.9–9.0)
	1 to < 5	827	48 (46–49)	70 (65–76)	2.0 (2.0–2.0)	2.5 (2.4–2.7)	4.1 (4.0–4.1)	5.1 (4.9–5.2)
	5 to < 10	1192	57 (55–59)	93 (88–102)	2.1 (2.1–2.2)	2.9 (2.8–3.1)	3.9 (3.8–4.0)	5.2 (5.0–5.5)
	10 to < 15	2066	122 (117–129)	210 (202–218)	3.8 (3.6–3.9)	5.8 (5.6–6.1)	5.9 (5.7–6.1)	8.9 (8.6–9.1)
	15 to < 18	2864	208 (202–215)	327 (313–339)	5.6 (5.4–5.7)	8.3 (8.1–8.6)	8.0 (7.8–8.2)	11.1 (10.9–11.4)
Abdomen and pelvis routine dose	< 1	269	68 (56–76)	102 (92–130)	2.5 (2.3–2.7)	3.3 (3.2–3.9)	5.7 (5.2–6.5)	7.6 (7.2–8.7)
	1 to < 5	1259	82 (79–85)	112 (108–117)	2.6 (2.5–2.6)	3.0 (3.0–3.1)	5.2 (5.0–5.3)	6.2 (6.0–6.3)
	5 to < 10	3425	126 (122–130)	199 (191–208)	3.3 (3.2–3.4)	4.7 (4.5–4.9)	6.0 (5.8–6.1)	8.2 (8.0–8.4)
	10 to < 15	6604	261 (257–264)	348 (343–354)	5.6 (5.5–5.6)	7.4 (7.2–7.5)	8.6 (8.5–8.7)	10.3 (10.2–10.5)
	15 to < 18	9041	335 (332–338)	495 (485–507)	6.7 (6.6–6.8)	9.9 (9.9–10.0)	9.5 (9.4–9.6)	12.8 (12.6–13.0)
Abdomen and pelvis high dose	< 1	3	–	–	–	–	–	–
	1 to < 5	13	151 (77–263)	263 (137–300)	1.9 (1.6–3.3)	3.3 (1.8–3.6)	5.9 (2.8–7.0)	6.8 (4.1–7.1)
	5 to < 10	24	234 (128–323)	343 (267–450)	3.2 (2.4–4.3)	4.4 (3.3–5.7)	5.9 (4.8–7.4)	7.7 (6.0–9.4)
	10 to < 15	54	558 (477–614)	762 (612–1004)	6.0 (5.5–6.2)	6.7 (6.2–11.5)	9.2 (8.6–9.6)	10.0 (9.6–14.2)
	15 to < 18	151	700 (641–834)	1100 (1003–1217)	6.5 (6.2–7.0)	9.9 (8.3–11.1)	9.7 (9.2–10.3)	12.4 (11.2–13.6)
Upper extremity routine dose	< 1	4	–	–	–	–	–	–
	1 to < 5	28	67 (48–83)	111 (73–144)	4.2 (3.3–5.0)	5.5 (4.3–9.8)	–	–
	5 to < 10	180	99 (82–117)	169 (153–182)	6.4 (5.2–7.5)	10.0 (9.4–10.2)	–	–
	10 to < 15	593	156 (144–170)	217 (201–231)	9.4 (8.3–9.4)	11.2 (10.2–11.9)	–	–
	15 to < 18	834	185 (176–192)	290 (272–304)	10.0 (10.0–10.1)	13.3 (12.8–14.3)	–	–
Lower extremity routine dose	< 1	7	–	–	–	–	–	–
	1 to < 5	24	70 (58–115)	139 (76–178)	3.0 (2.5–3.3)	3.9 (3.1–6.1)	–	–
	5 to < 10	192	121 (95–158)	216 (201–242)	5.2 (4.4–6.3)	10.0 (10.0–11.9)	–	–
	10 to < 15	1607	204 (196–210)	287 (281–295)	9.9 (9.8–9.9)	12.0 (12.0–12.1)	–	–
	15 to < 18	1336	230 (219–238)	309 (302–316)	9.9 (9.9–9.9)	12.0 (12.0–12.0)	–	–
Extremity high dose	< 1	2	–	–	–	–	–	–
	1 to < 5	1	–	–	–	–	–	–
	5 to < 10	5	–	–	–	–	–	–
	10 to < 15	19	511 (217–636)	740 (516–1025)	4.2 (2.9–12.8)	13.4 (4.9–20.5)	–	–
	15 to < 18	43	581 (475–838)	981 (730–1311)	6.8 (4.8–9.5)	16.2 (9.3–21.5)	–	–
Combined	< 1	59	58 (50–69)	97 (72–126)	2.1 (1.8–2.3)	2.6 (2.3–3.0)	4.8 (4.5–5.2)	5.9 (5.2–6.4)
	1 to < 5	331	110 (104–121)	164 (147–183)	2.6 (2.5–2.7)	3.3 (3.1–3.4)	5.1 (5.0–5.2)	6.1 (5.8–6.3)
	5 to < 10	391	170 (157–182)	233 (219–257)	3.1 (3.0–3.2)	4.2 (3.8–4.5)	5.6 (5.4–5.8)	7.0 (6.6–7.2)
	10 to < 15	515	358 (338–386)	552 (494–594)	5.5 (5.1–5.6)	7.5 (6.9–8.0)	8.4 (8.0–8.8)	11.0 (10.1–11.8)
	15 to < 18	911	612 (577–654)	1017 (955–1108)	7.1 (6.8–7.5)	11.4 (10.6–12.0)	10.0 (9.7–10.5)	15.2 (14.5–15.8)

Achievable doses (median) and DRLs for the different dose metrics (DLP, volumetric CT dose index, SSDE with 95%-confidence intervals (CI)) by age group

### Radiation dose and scan length of CT categories by age

**Fig. 1:**
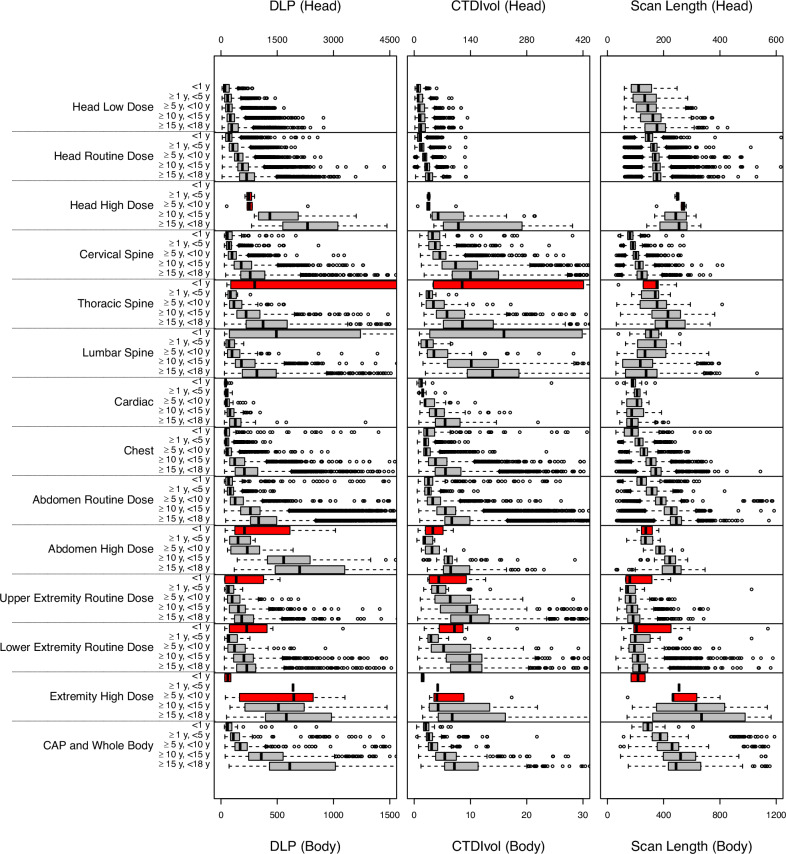
Distribution of radiation doses and scan length for CT categories by age group. Box plots show the distribution of DLP (in mGy·cm),  volumetric CT dose index (CTDI_vol_ in mGy), and scan length (in mm) for each CT category. Box edges indicate 25th and 75th percentiles. A thick vertical line indicates the median. Horizontal lines divide the CT categories. Red boxes indicate CT categories with a sample size of less than 10. Whiskers in the box plots refer to the maximum value no more than 1.5 interquartile ranges above the third quartile and the minimum value no more than 1.5 interquartile ranges below the first quartile

**Fig. 2:**
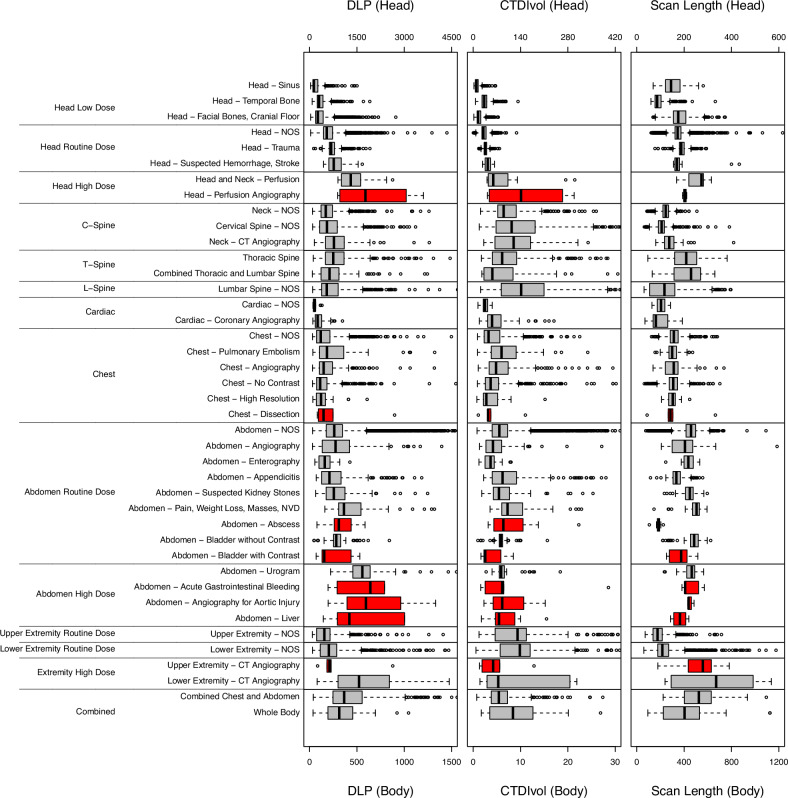
Distribution of radiation doses and scan length by different CT indications within CT categories for 10- to under 15-year-olds. Box plots show the distribution of DLP (in mGy·cm),  CTDI_vol_ (in mGy), and scan length (in mm) for each CT indication. Box edges indicate 25th and 75th percentiles. A thick vertical line indicates the median. Horizontal lines divide the CT categories. Red boxes indicate CT indications with a sample size of less than 10. Whiskers in the box plots refer to the maximum value no more than 1.5 interquartile ranges above the third quartile and the minimum value no more than 1.5 interquartile ranges below the first quartile

**Fig. 3:**
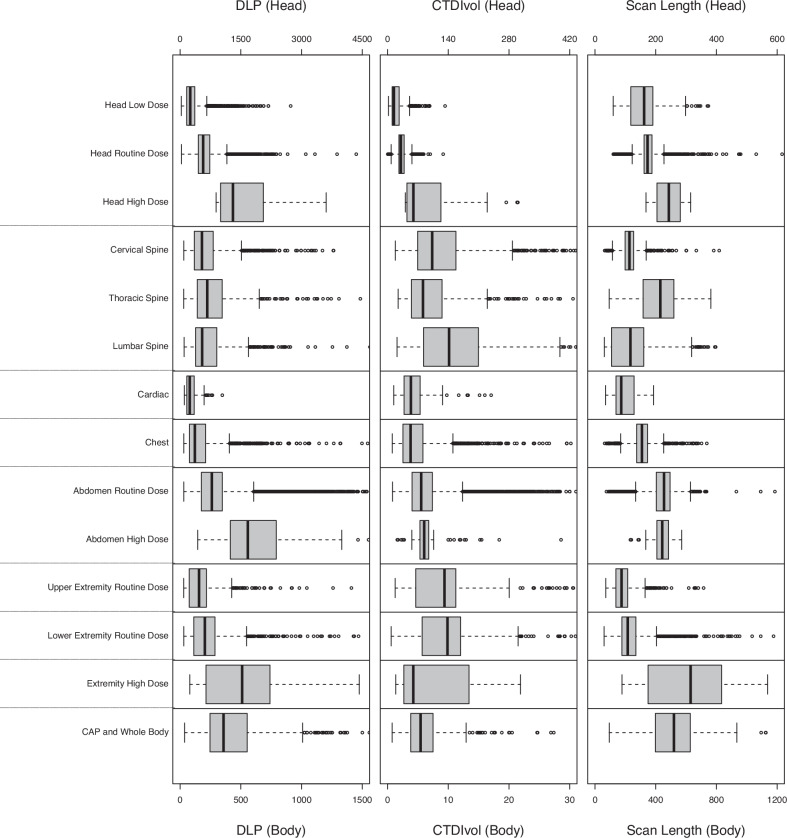
Distribution of radiation doses and scan length by CT categories for 10- to under 15-year-olds. Box plots show the distribution of DLP (in mGy·cm), CTDI_vol_ (in mGy), and scan length (in mm) for each CT category. Box edges indicate 25th and 75th percentiles. A thick vertical line indicates the median. Whiskers in the box plots refer to the maximum value no more than 1.5 interquartile ranges above the third quartile and the minimum value no more than 1.5 interquartile ranges below the first quartile

The dose metrics for each CT category are shown in Table 3, the distribution of radiation doses for CT categories by age group in Fig. 1, and by CT indication within categories for exemplary 10 to under 15 year old children in Fig. 2, and finally for the collapsed CT categories including the indications from Fig. 2 in Fig. 3 (all other age groups as Supplemental Figs. S1a–d and S2a–d). Obviously, radiation doses increase with increasing age group near monotonically across all CT categories with the exception of children under 1 year who have markedly elevated doses with wide confidence intervals for several categories with small sample sizes (Table 3 and Fig. 1). For example, for head low dose, the DRLs increased from 223 mGy·cm (for under 1 year) to 277 (1 to under 5-year-olds) to 298 (5 to under 10-year-olds), to 362 (10 to under 15- year- olds) to 461 mGy·cm (15 to under 18-year-olds), Table 3 and Fig. 1.

Within the body regions subdivided by dose (head, abdomen and pelvis, and extremities), the ADs and DRLs using DLP differed significantly between the CT categories. This can be seen by the fact that confidence intervals for ADs and DRL doses do not overlap between high, routine, and (in head) low dose categories within the same body region and age group (Table 3). For example, for head scans in 10- to under 15-year-olds, DRLs for the DLP increased from 362 mGy·cm for low dose to 734 mGy·cm for routine dose to 2058 mGy·cm for high dose (*p*-value < 0.001). For abdomen and pelvis scans, DRLs for the DLP increased from 348 mGy·cm for routine dose to 762 mGy·cm for high dose (*p* < 0.001), Table 3. For CTDI_vol_, this pronounced difference between dose categories is only seen in the head. For SSDE, this difference is not seen in the abdomen, the only body region for which the comparison is applicable.

Scan length varied by age within CT categories and by anatomic region; e.g., chest routine dose had a median scan length of 309 mm in 10- to under 15-year-olds compared with 173 mm for cardiac routine dose (Supplemental Table S1 and Fig. 2). However, the differences between adjacent age groups within CT categories were mostly not significant.

### Variation between CT categories

The variation was greater between CT categories in the head and for the oldest age group in the abdomen and pelvis than between indications within categories, meaning that the assignment of CT indications to the three CT categories of low, routine, and high dose (or routine and high in the abdomen and pelvis) was statistically supported, Supplemental Table S2.

### International comparison

**Fig. 4:**
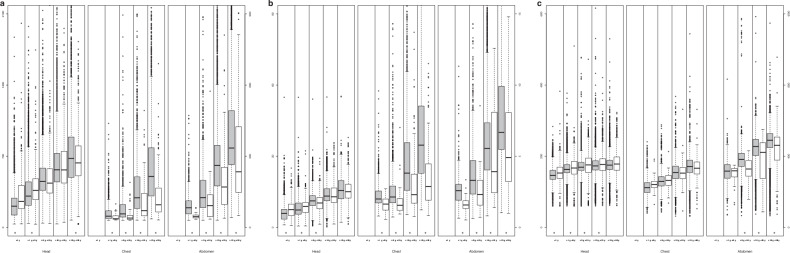
International comparison between the U.S. and European facilities within the registry. Box plots show the distribution of (**a**) DLP (in mGy·cm), (**b**) CTDI_vol_ (in mGy), and (**c**) scan length (in mm) for each CT category and age group. Box edges indicate 25th and 75th percentiles. A thick horizontal line indicates the median. Gray boxes represent the US data, and white boxes the European data. Samples less than 10 are not shown. Significant *p*-values (*p* < 0.05) for differences in median values are indicated by *. Whiskers in the box plots refer to the maximum value no more than 1.5 interquartile ranges above the third quartile and the minimum value no more than 1.5 interquartile ranges below the first quartile

Despite the U.S. having greater sample size, the most notable difference between U.S. and European facilities was that U.S. ADs and DRLs were statistically significantly higher for chest (relative U.S./European dose ratio 1.21–2.25) in all age groups and for nearly all doses for abdomen and pelvis (relative dose range 0.96–2.14, most statistically significantly different), Table 4. U.S. doses were lower for routine head CT for children under 5 years (relative dose range 0.70–0.97 across the different dose metrics and two age groups), Table 4 and Fig. 4a, b. Median scan length was significantly longer in European facilities than in the U.S. for head routine dose (except in the oldest age group), Fig. 4c.

**Table 4:** Radiation dose comparisons between facilities in the U.S. and Europe

CT category		Age group														
		< 1 year			1 to < 5 years			5 to < 10 years			10 to < 15 years			15 to < 18 years		
	Value	USA	Europe	Ratio USA/Europe	USA	Europe	Ratio USA/Europe	USA	Europe	Ratio USA/Europe	USA	Europe	Ratio USA/Europe	USA	Europe	Ratio USA/Europe
Head routine dose	No. of CT exams	5110	288		7421	582		6644	510		8335	537		8219	531	
	Median (50th) DLP	210	257	0.82*	309	364	0.85*	453	437	1.04	567	567	1.00	679	635	1.07*
	DRL (75th) DLP	288	412	0.70*	449	478	0.94*	584	577	1.01	728	750	0.97	889	802	1.11*
	Median (50th) CTDI_vol_	14	18	0.79*	17	21	0.83*	26	24	1.08*	31	30	1.02	36	35	1.03
	DRL (75th) CTDI_vol_	18	23	0.78*	24	25	0.97	31	30	1.06*	38	39	0.98	46	42	1.08*
Chest	No. of CT exams	225	3		787	26		1140	47		1888	127		2546	196	
	Median (50th) DLP	43	–	–	48	37	1.31*	57	40	1.46*	125	71	1.77*	215	96	2.25*
	DRL (75th) DLP	75	–	–	70	49	1.42*	96	51	1.89*	213	143	1.49*	336	166	2.03*
	Median (50th) CTDI_vol_	2.3	–	–	2.0	1.6	1.22*	2.1	1.6	1.37*	3.8	2.3	1.66*	5.8	2.9	2.01*
	DRL (75th) CTDI_vol_	3.6	–	–	2.6	2.0	1.29*	2.9	2.0	1.44*	6.0	3.7	1.59*	8.5	4.4	1.92*
	Median (50th) SSDE	4.8	–	–	4.1	3.4	1.21*	3.9	3.0	1.31*	6.0	3.7	1.65*	8.4	4.2	1.98*
	DRL (75th) SSDE	8.0	–	–	5.1	4.0	1.25*	5.3	3.7	1.41*	9.0	5.6	1.61*	11.4	6.7	1.70*
Abdomen/pelvis routine dose	No. of CT exams	260	6		1237	17		3368	21		6453	78		8745	137	
	Median (50th) DLP	68	–	–	83	46	1.82*	126	92	1.37*	262	171	1.53*	335	235	1.43*
	DRL (75th) DLP	100	–	–	112	53	2.14*	199	137	1.44	347	253	1.37	492	425	1.16*
	Median (50th) CTDI_vol_	2.5	–	–	2.6	1.6	1.63*	3.3	2.3	1.43*	5.6	3.9	1.42*	6.7	4.9	1.36*
	DRL (75th) CTDI_vol_	3.3	–	–	3.0	1.9	1.61*	4.7	3.3	1.42*	7.4	7.7	0.96	9.9	8.1	1.23*
	Median (50th) SSDE	5.5	–	–	5.2	3.3	1.56*	6.0	4.2	1.44*	8.6	5.4	1.57*	9.5	6.9	1.38*
	DRL (75th) SSDE	7.4	–	–	6.2	3.6	1.74*	8.2	5.5	1.48*	10.3	9.4	1.10	12.8	9.6	1.33

Radiation doses are given as median and DRL for the DLP (in mGy·cm), volumetric CT dose index (CTDI_vol_ in mGy), and SSDE (in mGy). Differences with significant *p*-values (*p* < 0.05) are indicated by *

## Discussion

We have provided dose metrics for pediatric CT by age and 14 broad CT categories that subdivide the two most common categories, head and abdomen and pelvis, into more granular categories that reflect underlying indications. We have shown that radiation dose varies meaningfully between these categories and that within these categories, it increases with age. These categories may be helpful for dose optimization and provide more granular guidance than the more broadly defined anatomic regions in other available benchmarks. Of note is that U.S. facilities in the Registry have up to twice the radiation dose as European facilities for routine dose chest and abdomen and pelvis CT, while these benchmarks are lower in the U.S. in younger children for head. Grouping multiple CT indications into broad CT categories that reflect different image quality requirements may facilitate radiation dose optimization and regulation. ADs and DRLs were mostly significantly different between CT categories in the same anatomic region, and analyses of variance support the validity of CT categories in the head for all age groups and in the abdomen and pelvis for older children. The high-dose abdomen and pelvis category is not commonly used in younger children. We created routine and high-dose extremity categories because of the large variation in scan length and radiation dose between routine indications and angiography, as well as two separate categories for routine dose of upper and lower extremities. With few exceptions, older age groups were associated with significantly higher radiation doses. This is mainly due to the growing body of the child, which is also reflected in the increasing diameter of the patient. Compared to previously published results in adults, the doses in adults are even higher than in the oldest age group of children (15 to under 18 years), with the exception of the head high dose, which may be due to the small sample size in children [20]. For example, from our 15 to under 18 years age group to adults in the Registry, the DLP DRLs for head increased as follows: 461 mGy·cm to 622 mGy·cm (head low dose) and 895 mGy·cm to 1050 mGy·cm (head routine dose) [20]. In international comparisons, U.S. facilities from the Registry had up to more than two times higher ADs and DRLs for chest and abdomen and pelvis routine dose. The value of these results must be considered preliminary because of the small number of contributing organizations and examinations from Europe. On the other hand, as far as our DRLs are comparable with pediatric DRLs published by the European Commission, our DRLs (mainly U.S. data) were slightly higher for chest imaging (chest routine dose) and on average lower for head imaging (head routine dose) [15]. For abdominal CT, the differences were inconsistent, with mainly equivalent values for CTDI_vol_, but some higher values for the DLP in children older than four years [15]. For chest and abdomen and pelvis comparisons, our age groups align closely with the most common age groups used for European DRLs. For head CT, while the European Commission groups all children > 6 years of age, we suggest additional age groups between 6 years and 18 years based on observed variation in this population.

Kanal et al published ADs and DRLs for the 10 most commonly performed pediatric CT examinations in the U.S. using the American College of Radiology (ACR) Dose Index Registry [6]. As far as age groups and CT categories with indications were comparable, our DRLs and ADs were mostly in line with these published values, with some differences. Overall, lower values were observed for our head routine dose DRLs compared to the head without contrast from the ACR. In addition, our doses for the head low dose were higher than for the sinuses without contrast; doses for our chest routine dose were higher than for the chest without contrast; and doses for our C-spine were lower than their cervical spine without contrast but equal to the doses for neck soft tissue with contrast. We were able to simplify these ten indications from the ACR to only six CT categories. Especially due to the problem of small sample sizes in children, it is helpful to group examinations into broader categories to obtain larger sample sizes and also to simplify the optimization process. Järvinen et al proposed exponential indication-based DRL curves, where dose metrics are plotted as a function of patient weight, as another approach to show benchmarks for CT radiation dose [25]. Almén et al suggested the use of weight-based diagnostic reference curves in clinics with low examination volumes where it is difficult to collect sufficient data for multiple weight groups [26].

The data analyzed and the ADs and DRLs presented have several advantages. First, they are comprehensive, especially for a pediatric radiology study, and include all examinations, not just selected patients as is often done to establish DRLs. In addition, they include a wide range of device makes and models and represent data from 143 imaging facilities worldwide. However, it should be noted that these data primarily reflect practice in the U.S. and were collected only from facilities that have invested in dose management software. Geographic regions other than the U.S., including Europe, have limited representation, and this limits international comparisons. Establishing DRLs by weight group, as recommended in the European guidelines for chest and abdomen and pelvis CT examination [15], was not possible in the Registry due to insufficient DICOM data for weight. However, we were able to add ADs and DRLs for the SSDE that also account for patient size. Alternatives would have been size-based DRLs in terms of water equivalent diameter [6, 27]. Finally, we decided to set the benchmarks by patient age group rather than by patient diameter for better clinical application (but diameters by age group are reported in Table S1). Another limitation is that the influence of techniques such as dose modulation and reconstruction algorithms on the radiation dose could not be analyzed.

It is of utmost importance to take measures to protect children from unnecessary exposure to ionizing radiation. Optimizing radiation dose in pediatric patients is a major challenge because radiation dose varies widely based on age, size, clinical indication, and protocol. Therefore, it is essential to analyze large data sets to gain insight into radiation dose in pediatric patients and to implement dose optimization strategies, such as DRLs. This work establishes achievable doses and DRLs for anatomy- and indication-based CT categories by age group in children using data from a large international dose registry. Future efforts by international committees such as ICRP should focus on ensuring and standardizing the recording of important parameters in DICOM tags, such as body weight in children, and the use of iterative or deep-learning-based reconstruction techniques. Developing algorithms for rapid DRL determination would further improve dose optimization and comparability.

## Supplementary information

ELECTRONIC SUPPLEMENTARY MATERIAL

## Abbreviations

CTDI_vol_: Volumetric CT dose index
DICOM: Digital imaging and communications in medicine
DLP: Dose-length product
DRL: Diagnostic reference level
NLP: Natural language processing
SSDE: Size-specific dose estimate
