# Supplementary material for: Diagnostic reference levels for indication-based CT categories in pediatric CT: data from an international registry
Source: Eur Radiol. 2025 Jun 14;35(12):7985–99. doi: 10.1007/s00330-025-11724-9 (PMC12634736; doi:10.1007/s00330-025-11724-9)
Supplement: Supplementary file 1 — ELECTRONIC SUPPLEMENTARY MATERIAL [file 330_2025_11724_MOESM1_ESM.pdf]

Diagnostic Reference Levels for Indication-Based CT Categories in Pediatric CT: Data from an International Registry

ELECTRONIC SUPPLEMENTARY MATERIAL

Table S1 Scan length and patient diameter by CT category and age group (all values are medians (IQR)).

CT Category	Age Group (years)	Patient Diameter (mm)	Scan Length (mm)
Head Low Dose	< 1	115 (98-128)	111 (85-157)
	1 - < 5	131 (115-143)	133 (91-174)
	5 - <10	141 (129-152)	144 (103-174)
	10 - < 15	153 (141-163)	162 (118-191)
	15 - < 18	158 (146-169)	176 (133-207)
Head Routine Dose	< 1	132 (122-139)	146 (135-161)
	1 - < 5	150 (144-155)	163 (154-177)
	5 - < 10	160 (155-164)	170 (160-184)
	10 - < 15	168 (163-173)	173 (162-188)
	15 - < 18	172 (166-177)	176 (163-190)
Head High Dose	< 1	-	-
	1 - < 5	-	-
	5 - < 10	-	-
	10 - < 15	177 (170-186)	243 (204-281)
	15 - < 18	197 (165-218)	255 (187-283)
C-Spine	< 1	117 (108-127)	160 (144-181)
	1 - < 5	125 (114-135)	181 (165-199)
	5 - < 10	134 (116-153)	202 (184-222)
	10 - < 15	138 (118-167)	227 (198-255)
	15 - < 18	146 (127-178)	245 (211-283)
T-Spine	< 1	-	-
	1 - < 5	111 (99-138)	341 (249-364)
	5 - < 10	129 (111-159)	353 (233-420)
	10 - < 15	159 (133-206)	431 (317-520)
	15 - < 18	160 (136-204)	422 (339-553)
L-Spine	< 1	153 (112-177)	309 (281-362)
	1 - < 5	113 (108-127)	340 (219-419)
	5 - < 10	140 (126-151)	267 (212-412)
	10 - < 15	144 (134-167)	234 (109-322)
	15 - < 18	159 (140-182)	275 (129-352)
Cardiac	< 1	104 (93-117)	181 (170-199)
	1 - < 5	126 (114-152)	211 (193-231)
	5 - < 10	132 (122-156)	211 (138-244)
	10 - < 15	162 (134-191)	173 (138-257)
	15 - < 18	152 (123-194)	173 (138-218)
Chest	< 1	126 (108-141)	173 (122-220)
	1 - < 5	157 (149-167)	225 (197-253)
	5 - < 10	186 (173-202)	259 (233-286)
	10 - < 15	234 (212-261)	309 (275-346)
	15 - < 18	257 (239-281)	344 (310-382)
Abdomen Routine Dose	< 1	134 (122-148)	241 (211-278)
	1 - < 5	158 (148-168)	317 (277-353)
	5 - < 10	187 (173-207)	382 (342-417)
	10 - < 15	236 (213-268)	455 (404-495)
	15 - < 18	256 (237-287)	490 (448-527)
Abdomen High Dose	< 1	-	-
	1 - < 5	163 (154-165)	270 (239-322)
	5 - < 10	180 (171-206)	372 (346-408)
	10 - < 15	242 (207-280)	443 (408-485)
	15 - < 18	255 (232-282)	476 (390-524)
Upper Extremity Routine Dose	< 1	-	-
	1 - < 5	57 (51-77)	144 (131-197)
	5 - < 10	76 (65-91)	163 (128-202)

Eur Radiol (2025) Bos D, Wang Y, Steward C, et al.

	10 - < 15	80 (60-115)	175 (138-215)
	15 - < 18	82 (61-150)	183 (145-231)
Lower Extremity Routine Dose	< 1		
	1 - < 5	78 (74-98)	198 (166-303)
	5 - < 10	93 (76-113)	192 (154-256)
	10 - < 15	100 (83-125)	216 (177-269)
	15 - < 18	111 (90-138)	228 (181-286)
Extremity High Dose	< 1	-	-
	1 - < 5	-	-
	5 - < 10	-	-
	10 - < 15	153 (100-184)	630 (350-834)
	15 - < 18	172 (140-217)	669 (322-979)
Combined	< 1	137 (119-145)	288 (253-319)
	1 - < 5	158 (148-167)	375 (319-428)
	5 - < 10	181 (171-198)	460 (354-507)
	10 - < 15	229 (206-252)	520 (398-628)
	15 - < 18	255 (232-279)	488 (434-664)

Table S2 Analyses of variance for CT indications and categories. If **B** greater than **A,** than CT categories are supported by the data (last column).

Body Region	CT Category	Age Group (years)	Variation between Indications within CT category				Variation between CT categories				
			Mean Square between Indications within CT Category	Mean Square within Indications	Between versus Within (F value)	Ratio of F value to N **(A)**	Mean Square between CT Categories within Body Regions	Mean Square within CT Category	Between versus Within (F value)	Ratio of F value to N **(B)**	If **B** greater than **A**
Head		< 1					28,602	1,824,554	64	0.011	
	High Dose		NA	NA	NA	NA					-
	Routine Dose		2,965,895	28,445	104	0.019					No
	Low Dose		94,244	19,230	5	0.015					No
		1 - < 5					41,264	18,716,629	454	0.049	
	High Dose		NA	NA	NA	NA					-
	Routine Dose		7,690,912	41,468	186	0.023					Yes
	Low Dose		864,875	32,064	27	0.023					Yes
		5 - < 10					50,290	105,414,678	2,096	0.210	
	High Dose		NA	NA	NA	NA					-
	Routine Dose		4,531,512	52,166	87	0.012					Yes
	Low Dose		5,899,933	39,353	150	0.055					Yes
		10 - < 15					76,938	136,953,301	1,780	0.142	
	High Dose		NA	NA	NA	NA					-
	Routine Dose		3,736,772	78,646	48	0.005					Yes
	Low Dose		9,606,032	62,388	154	0.044					Yes
		15 - < 18					103,393	186,455,734	1,803	0.148	
	High Dose		NA	NA	NA	NA					-
	Routine Dose		2,229,029	107,448	21	0.002					Yes
	Low Dose		13,639,493	76,956	177	0.056					Yes
Abdomen/Pelvis		15 - < 18					96,029	27,158,719	283	0.031	
	High Dose		587,885	337,016	1.7	0.012					Yes
	Routine Dose		1,739,326	90,680	19.2	0.002					Yes

NA - not available

Figure S1 Distribution of radiation doses and scan length by different CT indications within CT categories by age group. (**a**) for under 1-year-olds, **(b)** for 1- to under 5-year-olds, **(c)** for 5- to under 10-year-olds; **(d)** for 15- to under 18-year-olds. Box plots show distribution of dose-length product (DLP in mGy·cm) and volume CT dose index (CTDIvol in mGy), and scan length (in mm) for each CT indication. Box edges indicate 25th and 75th percentiles. Thick vertical line indicates median. Horizontal lines divide the CT categories. Red boxes indicate CT indications with a sample size less than 10. Whiskers in the box plots refer to the maximum value no more than 1.5 interquartile ranges above the third quartile and the minimum value no more than 1.5 interquartile ranges below the first quartile.

a

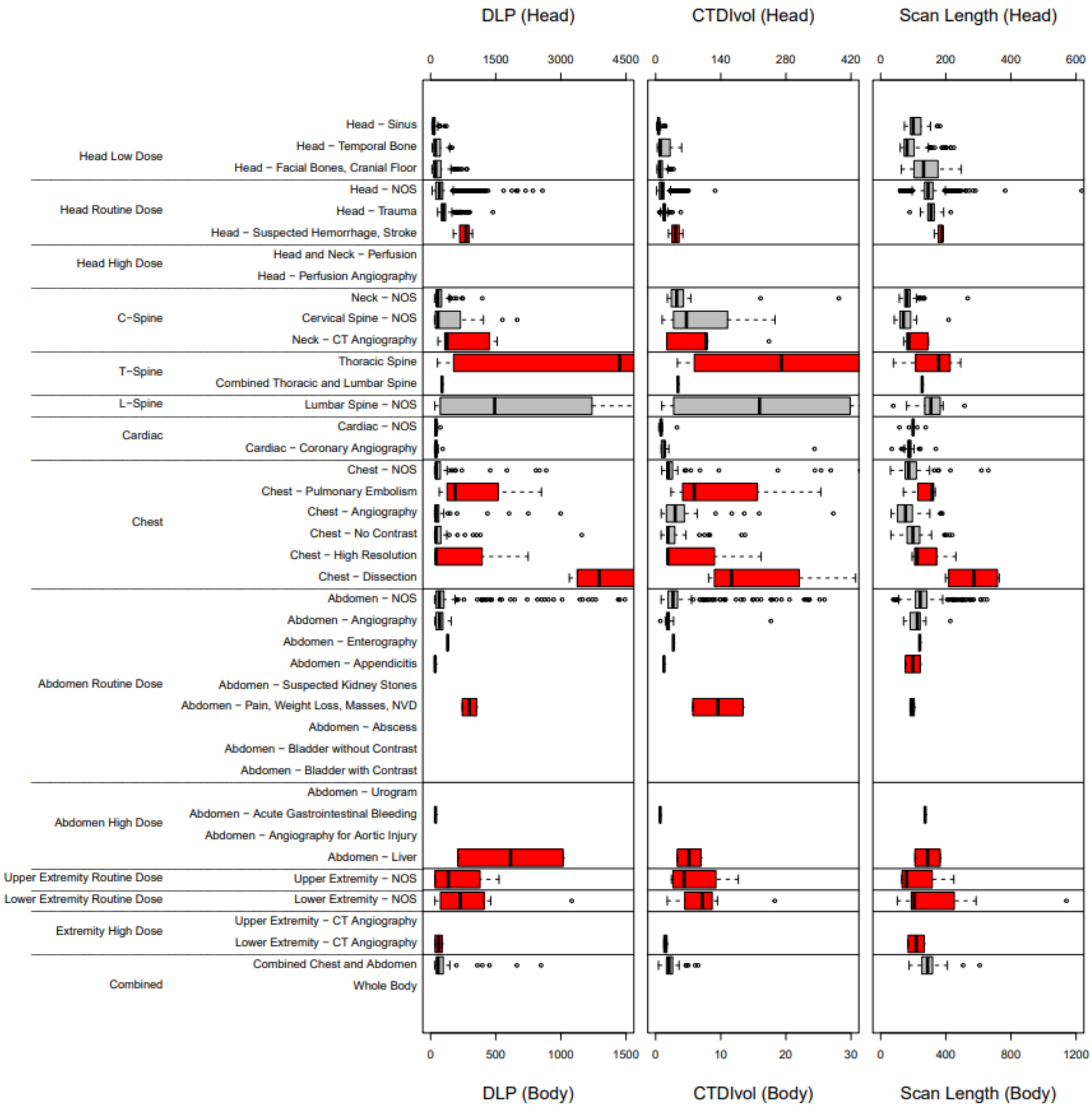

b

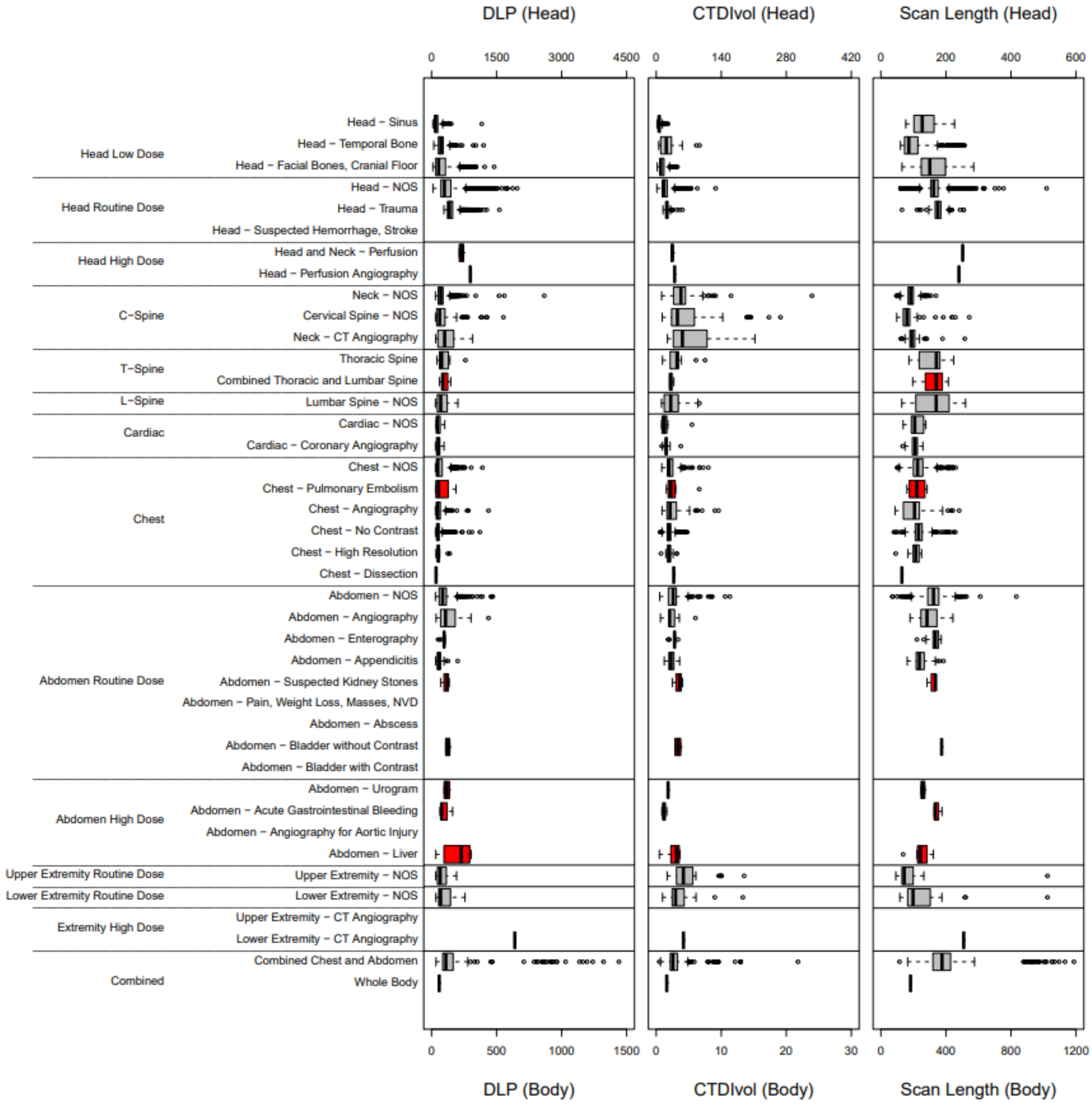
DLP (Head)
CTDIvol (Head)
Scan Length (Head)
0
1500
3000
4500
0
140
280
420
0
200
400
600
Head Low Dose
Head – Sinus
Head – Temporal Bone
Head – Facial Bones, Cranial Floor
Head Routine Dose
Head – NOS
Head – Trauma
Head – Suspected Hemorrhage, Stroke
Head High Dose
Head and Neck – Perfusion
Head – Perfusion Angiography
C–Spine
Neck – NOS
Cervical Spine – NOS
Neck – CT Angiography
T–Spine
Thoracic Spine
Combined Thoracic and Lumbar Spine
L–Spine
Lumbar Spine – NOS
Cardiac
Cardiac – NOS
Cardiac – Coronary Angiography
Chest
Chest – NOS
Chest – Pulmonary Embolism
Chest – Angiography
Chest – No Contrast
Chest – High Resolution
Chest – Dissection
Abdomen Routine Dose
Abdomen – NOS
Abdomen – Angiography
Abdomen – Enterography
Abdomen – Appendicitis
Abdomen – Suspected Kidney Stones
Abdomen – Pain, Weight Loss, Masses, NVD
Abdomen – Abscess
Abdomen – Bladder without Contrast
Abdomen – Bladder with Contrast
Abdomen High Dose
Abdomen – Urogram
Abdomen – Acute Gastrointestinal Bleeding
Abdomen – Angiography for Aortic Injury
Abdomen – Liver
Upper Extremity Routine Dose
Upper Extremity – NOS
Lower Extremity Routine Dose
Lower Extremity – NOS
Extremity High Dose
Upper Extremity – CT Angiography
Lower Extremity – CT Angiography
Combined
Combined Chest and Abdomen
Whole Body
0
500
1000
1500
0
10
20
30
0
400
800
1200
DLP (Body)
CTDIvol (Body)
Scan Length (Body)

c

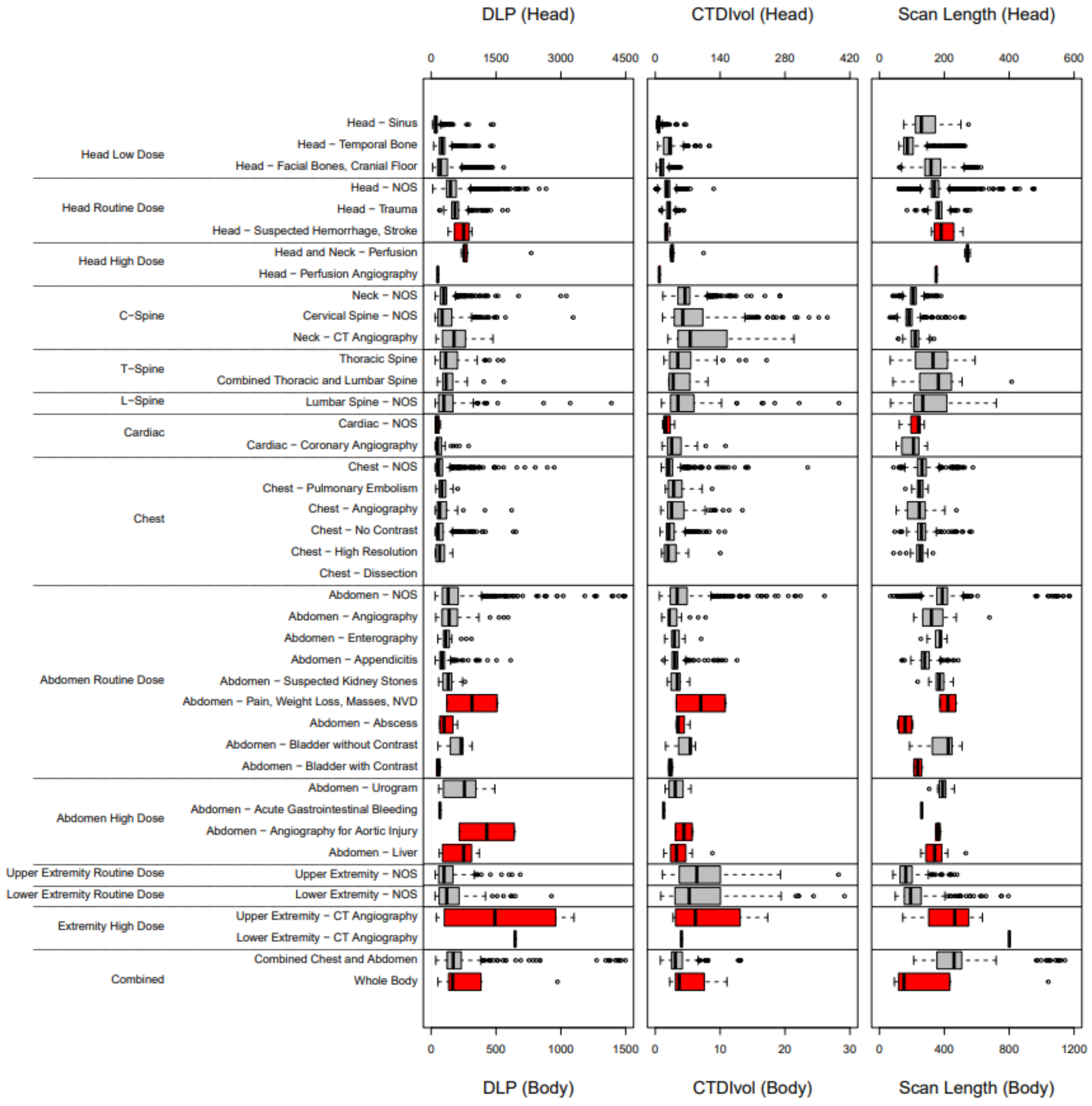

DLP (Head)
CTDIvol (Head)
Scan Length (Head)
0
1500
3000
4500
0
140
280
420
0
200
400
600
Head Low Dose
Head – Sinus
Head – Temporal Bone
Head – Facial Bones, Cranial Floor
Head Routine Dose
Head – NOS
Head – Trauma
Head – Suspected Hemorrhage, Stroke
Head High Dose
Head and Neck – Perfusion
Head – Perfusion Angiography
C–Spine
Neck – NOS
Cervical Spine – NOS
Neck – CT Angiography
T–Spine
Thoracic Spine
Combined Thoracic and Lumbar Spine
L–Spine
Lumbar Spine – NOS
Cardiac
Cardiac – NOS
Cardiac – Coronary Angiography
Chest
Chest – NOS
Chest – Pulmonary Embolism
Chest – Angiography
Chest – No Contrast
Chest – High Resolution
Chest – Dissection
Abdomen Routine Dose
Abdomen – NOS
Abdomen – Angiography
Abdomen – Enterography
Abdomen – Appendicitis
Abdomen – Suspected Kidney Stones
Abdomen – Pain, Weight Loss, Masses, NVD
Abdomen – Abscess
Abdomen – Bladder without Contrast
Abdomen – Bladder with Contrast
Abdomen High Dose
Abdomen – Urogram
Abdomen – Acute Gastrointestinal Bleeding
Abdomen – Angiography for Aortic Injury
Abdomen – Liver
Upper Extremity Routine Dose
Upper Extremity – NOS
Lower Extremity Routine Dose
Lower Extremity – NOS
Extremity High Dose
Upper Extremity – CT Angiography
Lower Extremity – CT Angiography
Combined
Combined Chest and Abdomen
Whole Body
0
500
1000
1500
0
10
20
30
0
400
800
1200
DLP (Body)
CTDIvol (Body)
Scan Length (Body)

d

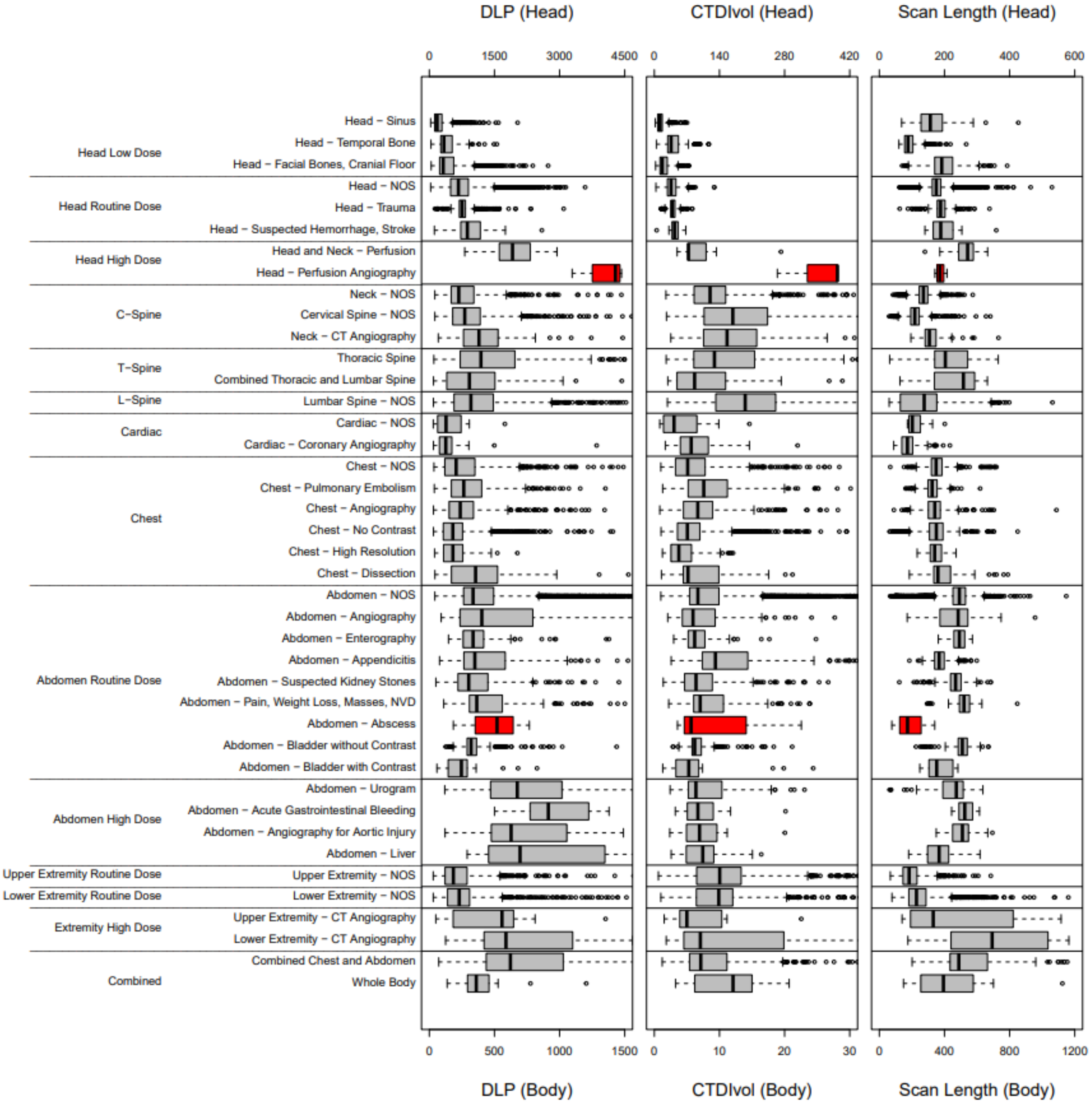

DLP (Head)
CTDIvol (Head)
Scan Length (Head)
0
1500
3000
4500
0
140
280
420
0
200
400
600
Head Low Dose
Head – Sinus
Head – Temporal Bone
Head – Facial Bones, Cranial Floor
Head Routine Dose
Head – NOS
Head – Trauma
Head – Suspected Hemorrhage, Stroke
Head High Dose
Head and Neck – Perfusion
Head – Perfusion Angiography
C–Spine
Neck – NOS
Cervical Spine – NOS
Neck – CT Angiography
T–Spine
Thoracic Spine
Combined Thoracic and Lumbar Spine
L–Spine
Lumbar Spine – NOS
Cardiac
Cardiac – NOS
Cardiac – Coronary Angiography
Chest
Chest – NOS
Chest – Pulmonary Embolism
Chest – Angiography
Chest – No Contrast
Chest – High Resolution
Chest – Dissection
Abdomen Routine Dose
Abdomen – NOS
Abdomen – Angiography
Abdomen – Enterography
Abdomen – Appendicitis
Abdomen – Suspected Kidney Stones
Abdomen – Pain, Weight Loss, Masses, NVD
Abdomen – Abscess
Abdomen – Bladder without Contrast
Abdomen – Bladder with Contrast
Abdomen High Dose
Abdomen – Urogram
Abdomen – Acute Gastrointestinal Bleeding
Abdomen – Angiography for Aortic Injury
Abdomen – Liver
Upper Extremity Routine Dose
Upper Extremity – NOS
Lower Extremity Routine Dose
Lower Extremity – NOS
Extremity High Dose
Upper Extremity – CT Angiography
Lower Extremity – CT Angiography
Combined
Combined Chest and Abdomen
Whole Body
0
500
1000
1500
0
10
20
30
0
400
800
1200
DLP (Body)
CTDIvol (Body)
Scan Length (Body)

Figure S2 Distribution of radiation doses and scan length by CT categories and age group. (a) for under 1-year-olds, **(b)** for 1- to under 5-year-olds, **(c)** for 5- to under 10-year-olds; **(d)** for 15- to under 18-year-olds. Box plots show distribution of dose-length product (DLP in mGy·cm) and volume CT dose index (CTDIvol in mGy), and scan length (in mm) for each CT category. Box edges indicate 25th and 75th percentiles. Thick vertical line indicates median. Red boxes indicate CT indications with a sample size less than 10. Whiskers in the box plots refer to the maximum value no more than 1.5 interquartile ranges above the third quartile and the minimum value no more than 1.5 interquartile ranges below the first quartile.

a

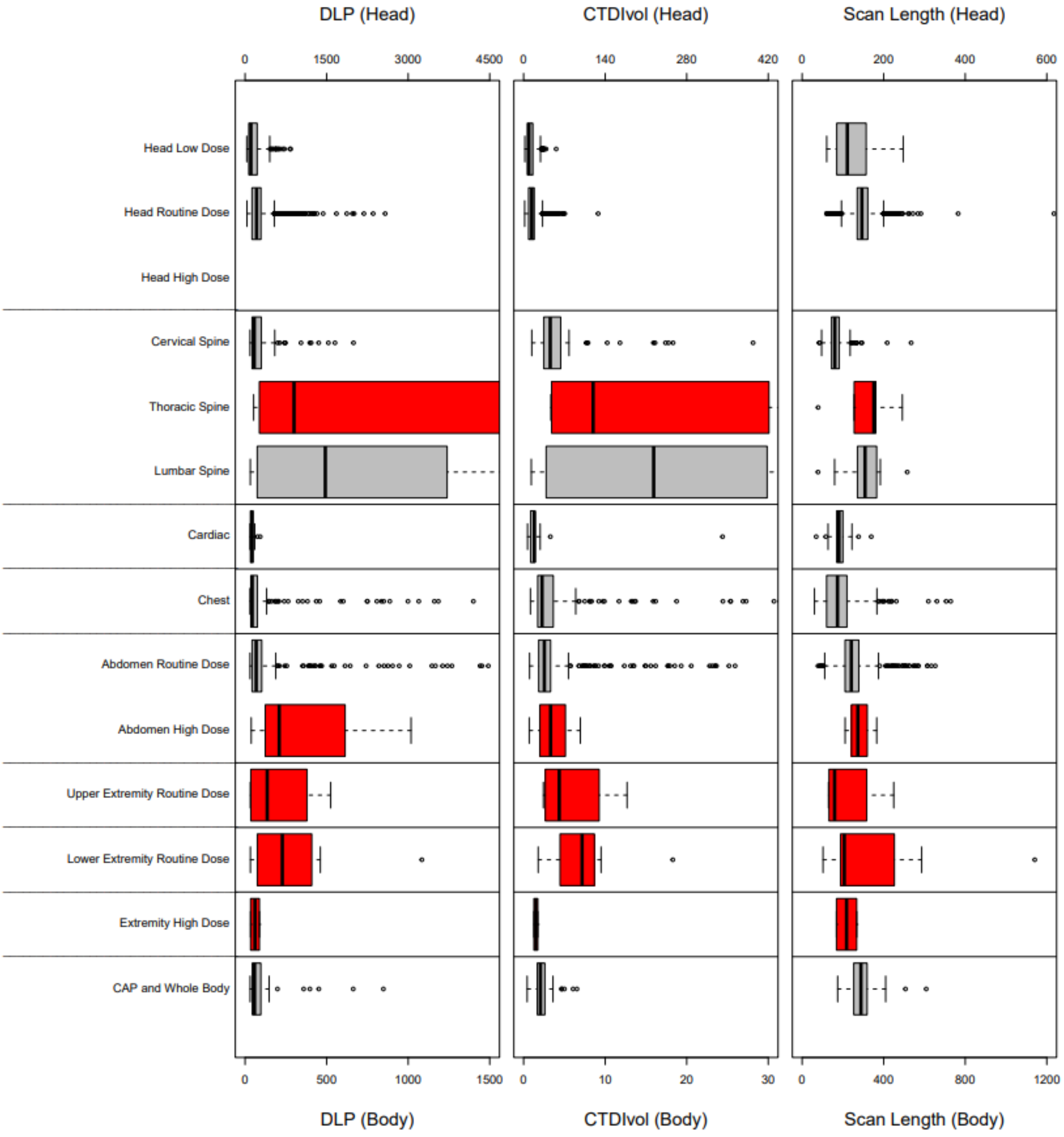

b

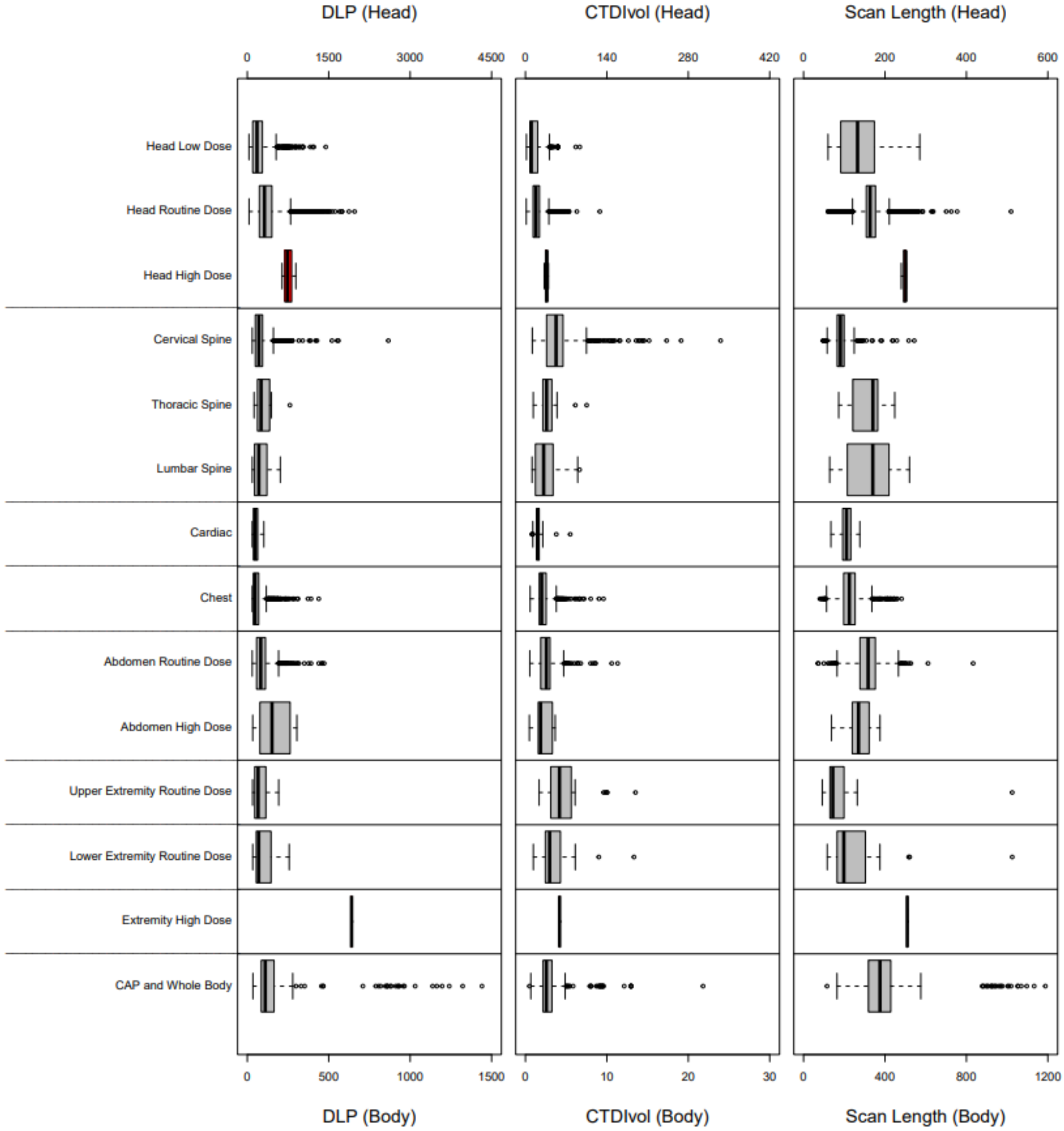

DLP (Head)
CTDIvol (Head)
Scan Length (Head)
0
1500
3000
4500
0
140
280
420
0
200
400
600
Head Low Dose
Head Routine Dose
Head High Dose
Cervical Spine
Thoracic Spine
Lumbar Spine
Cardiac
Chest
Abdomen Routine Dose
Abdomen High Dose
Upper Extremity Routine Dose
Lower Extremity Routine Dose
Extremity High Dose
CAP and Whole Body
0
500
1000
1500
0
10
20
30
0
400
800
1200
DLP (Body)
CTDIvol (Body)
Scan Length (Body)

c

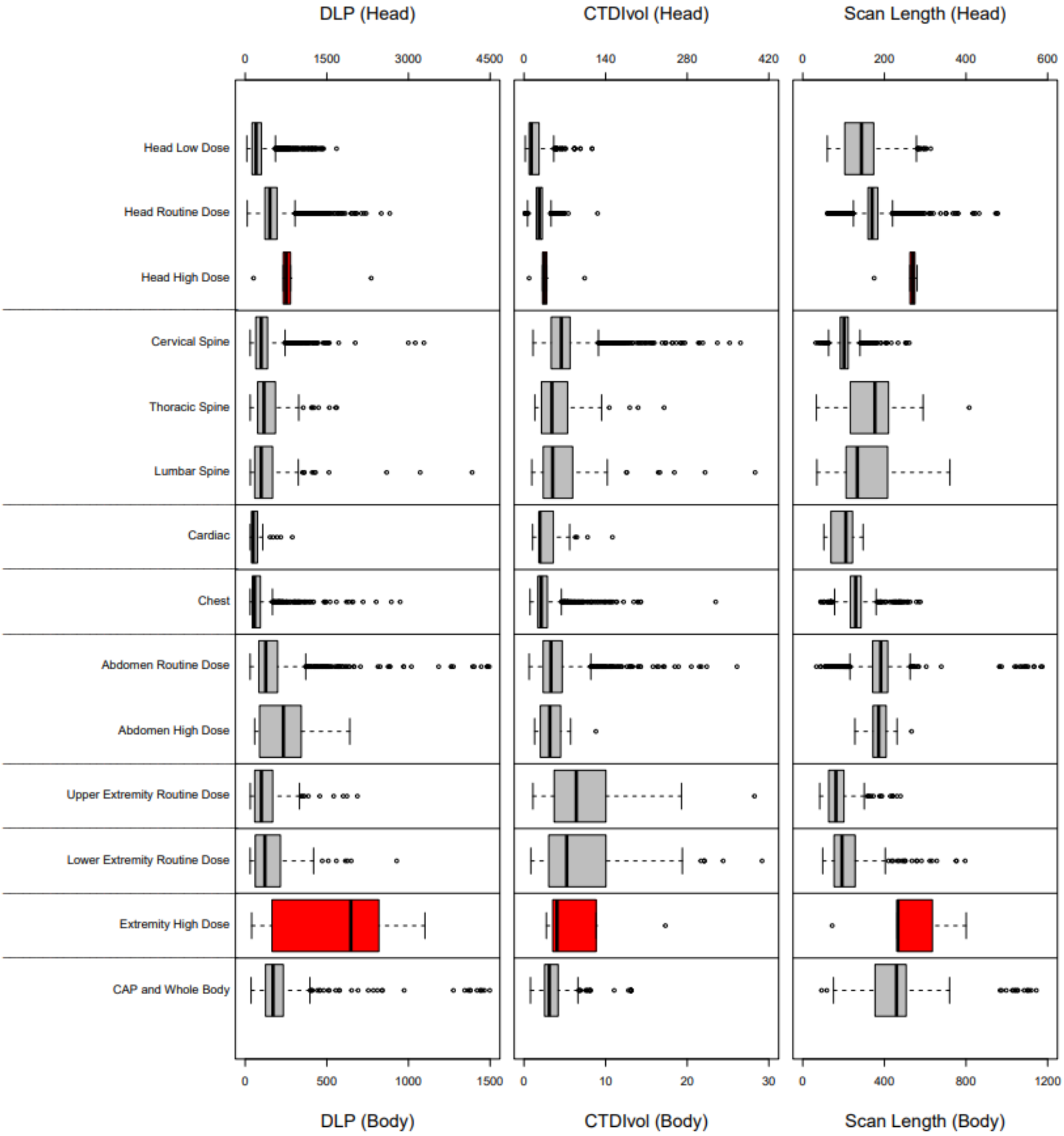
DLP (Head)
CTDIvol (Head)
Scan Length (Head)
0
1500
3000
4500
0
140
280
420
0
200
400
600
Head Low Dose
Head Routine Dose
Head High Dose
Cervical Spine
Thoracic Spine
Lumbar Spine
Cardiac
Chest
Abdomen Routine Dose
Abdomen High Dose
Upper Extremity Routine Dose
Lower Extremity Routine Dose
Extremity High Dose
CAP and Whole Body
0
500
1000
1500
0
10
20
30
0
400
800
1200
DLP (Body)
CTDIvol (Body)
Scan Length (Body)

d

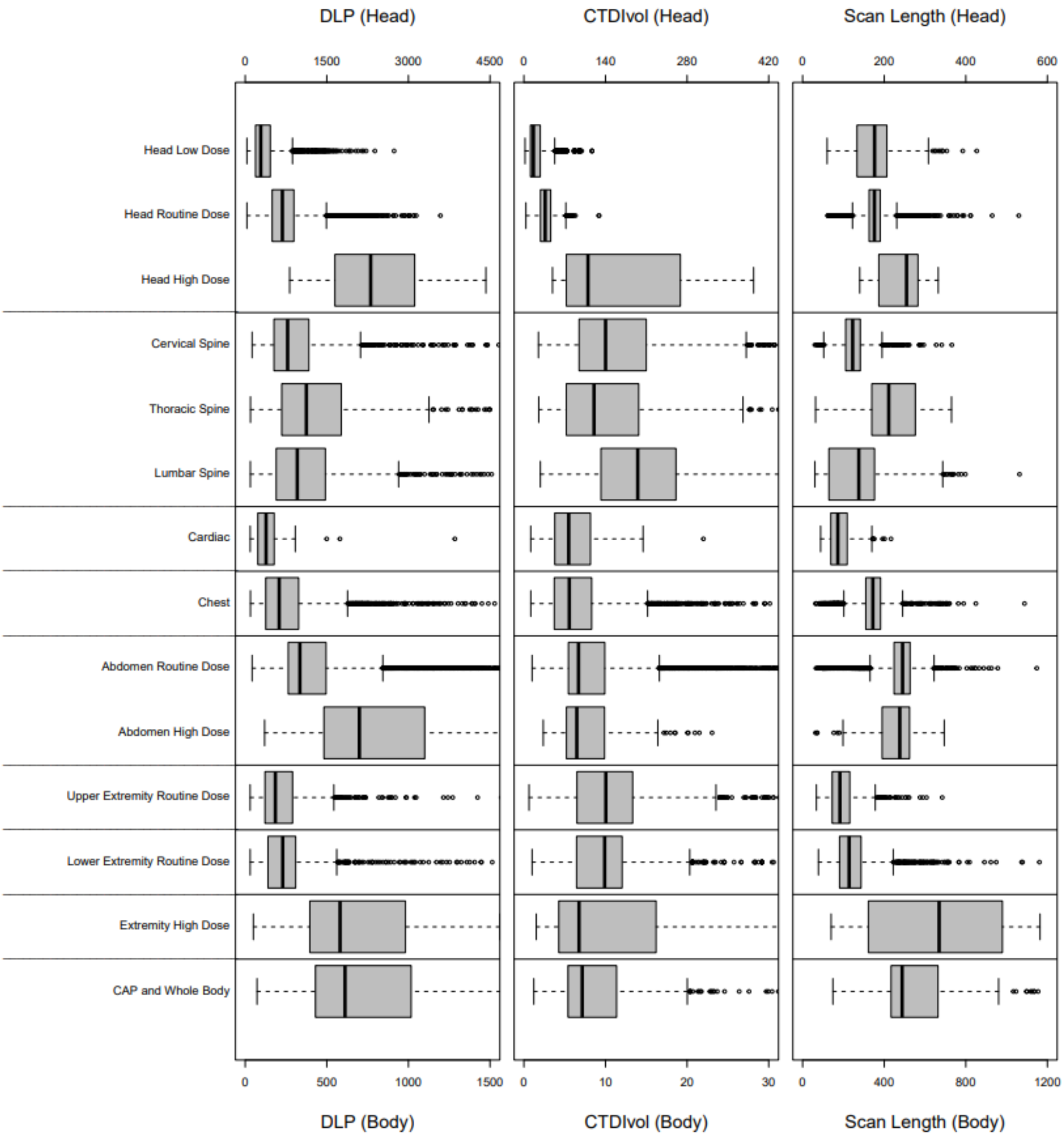
DLP (Head)
CTDIvol (Head)
Scan Length (Head)
0
1500
3000
4500
0
140
280
420
0
200
400
600
Head Low Dose
Head Routine Dose
Head High Dose
Cervical Spine
Thoracic Spine
Lumbar Spine
Cardiac
Chest
Abdomen Routine Dose
Abdomen High Dose
Upper Extremity Routine Dose
Lower Extremity Routine Dose
Extremity High Dose
CAP and Whole Body
0
500
1000
1500
0
10
20
30
0
400
800
1200
DLP (Body)
CTDIvol (Body)
Scan Length (Body)
